# Supplementary material for: Modeling glioblastoma heterogeneity as a dynamic network of cell states
Source: Mol Syst Biol. 2021 Sep 16;17(9):e10105. doi: 10.15252/msb.202010105 (PMC8444284; doi:10.15252/msb.202010105)
Supplement: Supplementary file 5 — Source Data for Figure 3 [file MSB-17-e10105-s001.zip › Figure3A_sourcedata/GSEA_3065/nosigdb_state6.GseaPreranked.1622718443993/gsea_report_for_na_pos_1622718443993.html]

Report for na\_pos 1622718443993 [GSEA]

| GS  follow link to MSigDB | GS DETAILS | SIZE | ES | NES | NOM p-val | FDR q-val | FWER p-val | RANK AT MAX | LEADING EDGE || 1 | NB\_KI\_CLUSTER1 | Details ... | 32 | 0.94 | 3.15 | 0.000 | 0.000 | 0.000 | 255 | tags=94%, list=3%, signal=96% |
| 2 | LARSSON\_STATE\_6 | Details ... | 101 | 0.65 | 2.75 | 0.000 | 0.000 | 0.000 | 1403 | tags=66%, list=16%, signal=78% |
| 3 | SUVA\_NPC1\_PMID\_31327527 | Details ... | 34 | 0.69 | 2.36 | 0.000 | 0.000 | 0.000 | 942 | tags=47%, list=11%, signal=52% |
| 4 | RICHARDS\_NEFTEL\_CELL\_2019\_NPC1 | Details ... | 34 | 0.69 | 2.35 | 0.000 | 0.000 | 0.000 | 942 | tags=47%, list=11%, signal=52% |
| 5 | RICHARDS\_NOWAKOWSKI\_SCIENCE\_2017\_IPC\_NEN3\_UPREG | Details ... | 227 | 0.47 | 2.22 | 0.000 | 0.000 | 0.002 | 857 | tags=30%, list=10%, signal=32% |
| 6 | OLSEN\_ADRENERGIC | Details ... | 64 | 0.52 | 2.01 | 0.000 | 0.001 | 0.011 | 807 | tags=41%, list=9%, signal=44% |
| 7 | NB\_KI\_CLUSTER4 | Details ... | 90 | 0.46 | 1.96 | 0.000 | 0.001 | 0.013 | 1649 | tags=42%, list=19%, signal=51% |
| 8 | RICHARDS\_VERHAAK\_CANCERCELL\_2010\_PRONEURAL | Details ... | 107 | 0.43 | 1.87 | 0.000 | 0.005 | 0.049 | 1090 | tags=26%, list=12%, signal=30% |
| 9 | RICHARDS\_NOWAKOWSKI\_SCIENCE\_2017\_MGE\_RG2\_UPREG | Details ... | 435 | 0.37 | 1.87 | 0.000 | 0.004 | 0.050 | 863 | tags=25%, list=10%, signal=26% |
| 10 | SUVA\_NPC2\_PMID\_31327527 | Details ... | 32 | 0.53 | 1.80 | 0.000 | 0.006 | 0.078 | 1649 | tags=44%, list=19%, signal=54% |
| 11 | RICHARDS\_NOWAKOWSKI\_SCIENCE\_2017\_RG\_EARLY\_UPREG | Details ... | 430 | 0.35 | 1.80 | 0.000 | 0.006 | 0.084 | 814 | tags=23%, list=9%, signal=24% |
| 12 | RICHARDS\_NOWAKOWSKI\_SCIENCE\_2017\_IPC\_NEN1\_UPREG\_1 | Details ... | 131 | 0.40 | 1.79 | 0.000 | 0.005 | 0.087 | 1267 | tags=28%, list=14%, signal=33% |
| 13 | RICHARDS\_NEFTEL\_CELL\_2019\_NPC2 | Details ... | 32 | 0.53 | 1.77 | 0.000 | 0.007 | 0.116 | 1649 | tags=44%, list=19%, signal=54% |
| 14 | RICHARDS\_NOWAKOWSKI\_SCIENCE\_2017\_NIN4\_UPREG | Details ... | 131 | 0.40 | 1.77 | 0.000 | 0.006 | 0.119 | 1213 | tags=31%, list=14%, signal=36% |
| 15 | WENG\_STEMNESS\_PMID\_30982771 | Details ... | 35 | 0.51 | 1.73 | 0.007 | 0.009 | 0.167 | 1346 | tags=51%, list=15%, signal=60% |
| 16 | RICHARDS\_NOWAKOWSKI\_SCIENCE\_2017\_MGE\_IPC3\_UPREG | Details ... | 114 | 0.38 | 1.64 | 0.002 | 0.017 | 0.321 | 1213 | tags=32%, list=14%, signal=37% |
| 17 | RICHARDS\_NOWAKOWSKI\_SCIENCE\_2017\_NIN1\_UPREG | Details ... | 146 | 0.34 | 1.54 | 0.000 | 0.037 | 0.579 | 1353 | tags=29%, list=15%, signal=33% |
| 18 | DOROSZKO\_U3180MG\_CLUSTER\_6 | Details ... | 39 | 0.40 | 1.44 | 0.055 | 0.072 | 0.827 | 1449 | tags=38%, list=16%, signal=46% |
| 19 | RICHARDS\_NOWAKOWSKI\_SCIENCE\_2017\_NIN3\_UPREG | Details ... | 137 | 0.31 | 1.38 | 0.016 | 0.108 | 0.940 | 981 | tags=22%, list=11%, signal=24% |
| 20 | RICHARDS\_NOWAKOWSKI\_SCIENCE\_2017\_IN\_CTX\_MGE2\_UPREG | Details ... | 193 | 0.29 | 1.37 | 0.015 | 0.106 | 0.946 | 1407 | tags=26%, list=16%, signal=30% |
| 21 | RICHARDS\_VERHAAK\_CANCERCELL\_2010\_NEURAL |  | 57 | 0.36 | 1.37 | 0.064 | 0.104 | 0.949 | 1094 | tags=23%, list=12%, signal=26% |
| 22 | GAROFANO\_IAVARONE\_MTC\_SUBTYPE |  | 207 | 0.28 | 1.35 | 0.006 | 0.114 | 0.964 | 1539 | tags=23%, list=17%, signal=27% |
| 23 | RICHARDS\_NOWAKOWSKI\_SCIENCE\_2017\_IPC\_NEN1\_UPREG |  | 221 | 0.27 | 1.30 | 0.018 | 0.147 | 0.989 | 1234 | tags=21%, list=14%, signal=24% |
| 24 | RICHARDS\_NOWAKOWSKI\_SCIENCE\_2017\_IN\_CTX\_MGE1\_UPREG |  | 276 | 0.27 | 1.29 | 0.019 | 0.147 | 0.991 | 1377 | tags=24%, list=16%, signal=28% |
| 25 | RICHARDS\_NOWAKOWSKI\_SCIENCE\_2017\_IN\_CTX\_CGE1\_UPREG |  | 294 | 0.25 | 1.25 | 0.024 | 0.181 | 0.996 | 2003 | tags=30%, list=23%, signal=37% |
| 26 | VANGRONINGEN\_ADRN |  | 224 | 0.26 | 1.25 | 0.043 | 0.185 | 0.996 | 1689 | tags=28%, list=19%, signal=34% |
| 27 | GAROFANO\_IAVARONE\_NEU\_SUBTYPE |  | 153 | 0.27 | 1.24 | 0.081 | 0.188 | 0.997 | 1663 | tags=23%, list=19%, signal=28% |
| 28 | NB\_KI\_CLUSTER2 |  | 249 | 0.25 | 1.22 | 0.075 | 0.197 | 0.999 | 1462 | tags=28%, list=17%, signal=32% |
| 29 | DOROSZKO\_U3013MG\_CLUSTER\_3 |  | 99 | 0.29 | 1.21 | 0.128 | 0.202 | 1.000 | 1515 | tags=36%, list=17%, signal=43% |
| 30 | TIISIG\_PMID\_32649888 |  | 22 | 0.39 | 1.21 | 0.195 | 0.204 | 1.000 | 70 | tags=14%, list=1%, signal=14% |
| 31 | RICHARDS\_NOWAKOWSKI\_SCIENCE\_2017\_NIN5\_UPREG |  | 253 | 0.24 | 1.13 | 0.152 | 0.303 | 1.000 | 1234 | tags=21%, list=14%, signal=24% |
| 32 | ROSEN\_KRONA\_TI\_UNPUBL |  | 46 | 0.30 | 1.11 | 0.251 | 0.332 | 1.000 | 1498 | tags=26%, list=17%, signal=31% |
| 33 | RICHARDS\_NOWAKOWSKI\_SCIENCE\_2017\_NEN\_LATE\_UPREG |  | 351 | 0.22 | 1.09 | 0.190 | 0.378 | 1.000 | 769 | tags=12%, list=9%, signal=13% |
| 34 | IVY\_IT |  | 31 | 0.32 | 1.07 | 0.335 | 0.408 | 1.000 | 903 | tags=29%, list=10%, signal=32% |
| 35 | NB\_KI\_CLUSTER3 |  | 160 | 0.23 | 1.05 | 0.301 | 0.428 | 1.000 | 1085 | tags=17%, list=12%, signal=19% |
| 36 | NB\_KI\_CLUSTER13 |  | 109 | 0.24 | 1.03 | 0.341 | 0.468 | 1.000 | 750 | tags=15%, list=9%, signal=16% |
| 37 | RICHARDS\_NOWAKOWSKI\_SCIENCE\_2017\_EN\_PFC3\_UPREG |  | 193 | 0.21 | 1.01 | 0.428 | 0.516 | 1.000 | 780 | tags=13%, list=9%, signal=14% |
| 38 | NB\_KI\_CLUSTER22 |  | 127 | 0.22 | 0.97 | 0.511 | 0.589 | 1.000 | 1893 | tags=27%, list=22%, signal=34% |
| 39 | SINGH\_SOX2\_UPREGULATED\_TRANSCRIPTS\_PMID\_28122245 |  | 405 | 0.19 | 0.95 | 0.668 | 0.645 | 1.000 | 1286 | tags=18%, list=15%, signal=20% |
| 40 | RICHARDS\_NOWAKOWSKI\_SCIENCE\_2017\_NEN\_EARLY2\_UPREG |  | 382 | 0.18 | 0.93 | 0.793 | 0.687 | 1.000 | 1734 | tags=23%, list=20%, signal=27% |
| 41 | GANDAL\_SCHIZOPHRENIA\_DN\_PMID29439242 |  | 16 | 0.32 | 0.91 | 0.590 | 0.713 | 1.000 | 924 | tags=19%, list=11%, signal=21% |
| 42 | NB\_KI\_CLUSTER16 |  | 71 | 0.22 | 0.90 | 0.636 | 0.718 | 1.000 | 1145 | tags=18%, list=13%, signal=21% |
| 43 | DOROSZKO\_U3054MG\_CLUSTER\_7 |  | 27 | 0.27 | 0.86 | 0.654 | 0.790 | 1.000 | 1248 | tags=22%, list=14%, signal=26% |
| 44 | NB\_KI\_CLUSTER12 |  | 36 | 0.19 | 0.65 | 0.947 | 0.977 | 1.000 | 4 | tags=3%, list=0%, signal=3% |
Table: Gene sets enriched in phenotype **na **[plain text format]****
